# How CEO Workaholism Influences Firm Performance: The Roles of Collective Organizational Engagement and TMT Power Distance

**DOI:** 10.3389/fpsyg.2021.725199

**Published:** 2021-09-21

**Authors:** Zhuolin She, Quan Li, Jilei Zhou

**Affiliations:** ^1^School of Public Administration and Policy, Renmin University of China, Beijing, China; ^2^Business School, Nankai University, Tianjin, China; ^3^School of Information, Renmin University of China, Beijing, China

**Keywords:** CEO workaholism, collective organizational engagement, TMT power distance, firm performance, upper echelons theory

## Abstract

Based on upper echelons theory, the current study examines how and under what conditions CEO workaholism influences firm performance. Specifically, we propose that CEO workaholism is positively related to collective organizational engagement, which has a subsequent positive effect on firm performance. Top management team (TMT) power distance would moderate the relationship between CEO workaholism and collective organizational engagement in such a way that workaholic CEOs are more likely to stimulate collective organizational engagement when TMTs have a high level of power distance. Findings based on multi-source, multi-wave data from a sample of 122 CEOs in state-owned enterprises and their corresponding TMT members supported the hypotheses. This study is an initial attempt to empirically examine the effects of leader workaholism at the firm level, which answers the call for more research into the intersection of workaholism and leadership and carries implications for organizational management practices.

## Introduction

Over the past decades, the intense global competitiveness has made workers increasingly exposed to demanding working conditions. Moreover, with the advancement in communication technology, people can frequently connect to work outside the traditional office and traditional work hours (Ng et al., 2007). These changes inevitably lead to longer working hours and induce more workaholic behaviors (Clark et al., 2016a). Workaholism is typically described as “the tendency to work excessively hard and being obsessed with work” (Schaufeli et al., 2009, p. 322). Generally, previous studies have shown that workaholism is primarily linked to unfavorable outcomes (see Clark et al., 2016a for a recent meta-analysis), such as reduced job satisfaction (Dordoni et al., 2019), increased job burnout (Schaufeli et al., 2008), decreased work-related health (Langseth-Eide, 2019), and spouses' greater marital estrangement (Robinson et al., 2001). However, recently, researchers have questioned the prevailing belief that workaholism is necessarily bad, suggesting that it can positively affect employees. For example, Ng et al. (2007) have hinted at possible positive effects of workaholism, such as increased productivity and career success.

Despite efforts devoted to understanding the outcomes of workaholism in organizations, there are some major limitations in this literature. First, a dearth of studies has investigated how leader workaholism impacts their employees (see Clark et al., 2016b; Pan, 2018 for possible exceptions). This seems an important omission given that leader plays an important role in shaping employees' workplace perceptions and behaviors (Yukl, 2002). With the prevalence of workaholic leaders in the workplace (Brett and Stroh, 2003; Knight, 2016), a better understanding of the effects of leader workaholism is needed. Second, in focusing primarily on the negative consequences of workaholism, scholars have largely overlooked the possibility that leader workaholism may have advantages (Clark et al., 2016b). Third, prior research has mostly centered on the influence of workaholism at the individual level while ignoring its effects at the firm level. As CEOs attributes have important influences on organizational outcomes (Hambrick and Mason, 1984; Shah et al., 2021), the connection between CEO workaholism and firm performance is deserving of more attention. Accordingly, we aim to fill these research gaps by exploring whether and how CEO workaholism would affect firm performance, thus extending the line of workaholism research that previously has not been fully considered.

**Figure 1:**
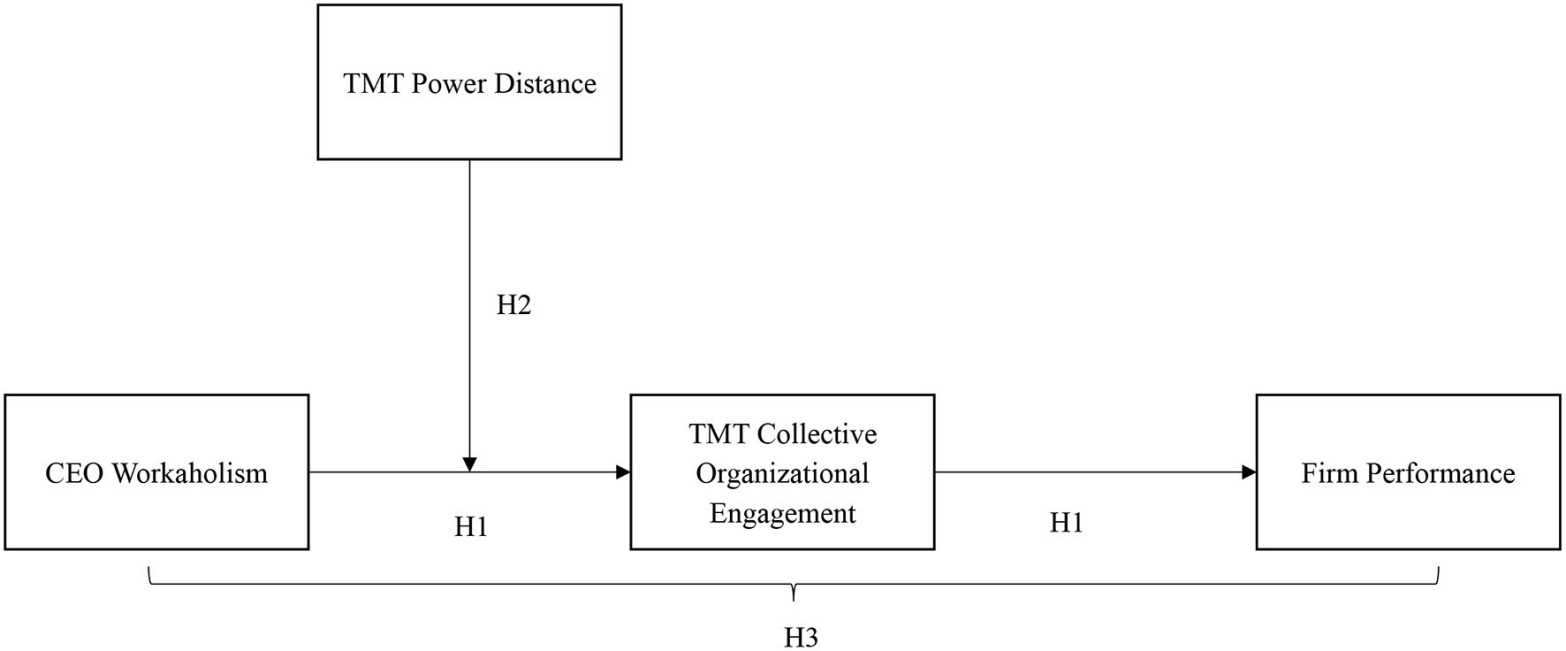
The proposed model.

According to upper echelons theory (Hambrick and Mason, 1984; Hambrick and Finkelstein, 1996), CEO attributes exert significant impacts on firm outcomes. Moreover, the relationship between CEOs attributes and firm outcomes is inevitably transmitted through TMT members. As such, we expect that top management teams' (TMTs) collective organizational engagement is a crucial TMT functioning linking workaholic CEOs and firm performance. Collective organizational engagement of TMTs refers to the degree to which TMT members invest themselves in their work (Barrick et al., 2015). Under the influence of workaholic CEOs, TMT members are likely to be motived to exert efforts at work and to perform beyond expectations, thus fostering TMTs' collective organizational engagement and ultimately exerting a salient influence on overall firm performance. In addition, upper echelons theory also posits that TMT characteristics would influence the strength of CEO attributes' effects (Hambrick and Mason, 1984; Hambrick and Finkelstein, 1996). From a CEO-TMT interface perspective, we thus incorporate TMT power distance (the extent to which TMT members accept unequal distribution of power, Farh et al., 2007) into the model and argue that the positive effects of CEO workaholism on collective organizational engagement will be stronger when TMTs have a high level of power distance. The proposed model is shown in Figure 1.

By testing this model in a time-lagged multisource study of CEO-TMT member dyads from state-owned enterprises, we make several contributions. First, we extend the nomological network of the consequences of workaholism by testing the effects of CEO workaholism on firm performance, answering the call for more research into the effects of leader workaholism (Clark et al., 2016b). Second, based on upper echelons theory, we identify TMT collective organizational engagement as an important mediating mechanism that connects CEO workaholism to firm performance. This is an initial attempt to theorize explicitly and empirically examine the process of TMT dynamics by which CEO workaholism influences firm performance. Finally, our consideration of TMT power distance adds to a comprehensive understanding of the boundary conditions under which CEO workaholism influences firm outcomes.

## Theoretical Background and Hypotheses Development

### Workaholism in Organizations

Though there is still much debate over the precise conceptualization and measurement of workaholism (Ng et al., 2007; Clark et al., 2016a), researchers have generally described it as one's uncontrollable addictive tendency to be preoccupied with work and to work beyond expectations (Spence and Robbins, 1992; Robinson, 1999; Balducci et al., 2018). Schaufeli et al. (2008) further proposed that the construct of workaholism contains two elements, namely working excessively (exceptional amount of time and energy that workaholics devote to work activities) and work compulsively (a strong and irresistible inner drive to work).

Accordingly, previous studies have highlighted three common characteristics of workaholics (Scott et al., 1997; Snir and Harpaz, 2012). First, workaholics have a strong internal drive. They are excessively obsessed with their work. This obsessive drive mainly comes from internal forces rather than from external or contextual factors, such as money, rewards, and other external stimuli (McMillan and O'Driscoll, 2006). Second, workaholics spend a great deal of time and energy on work, far beyond what is reasonably expected by their organizations. Third, workaholics are unwilling to disengage themselves from work and constantly think about work even when not working (Yang et al., 2020).

Since individuals holding managerial roles particularly exhibit workaholic behaviors (Brett and Stroh, 2003; Friedman and Lobel, 2003), researchers recently applied the workaholism definition to the leadership context and suggested that leaders are particularly likely to experience and exhibit the cognitive and behavioral aspects of workaholism (Pan, 2018; Li and She, 2020). Consistent with existing research, in the current study, we focus on CEO workaholism, and describe it as a CEO's addition to work, manifesting as an inner drive to work compulsively and excessively (Clark et al., 2016b; Pan, 2018; Li and She, 2020).

### CEO Workaholism, Collective Organizational Engagement and Firm Performance

As suggested by upper echelons theory (Hambrick and Finkelstein, 1996), a CEO does not wield influence in isolation from TMT members. Specifically, as CEOs frequently interact with TMT members, their individual attributes can significantly impact TMT processes, which in turn are related to firm outcomes (Peterson et al., 2003; Shah et al., 2019). In this case, we posit that TMTs' collective organizational engagement plays an important mediating role in the relationship between workaholic CEOs and firm performance. Collective organizational engagement is defined as the extent to organizations' members cognitively, emotionally, and physically invest in their work (Barnes et al., 2015; Barrick et al., 2015). We expect that CEO workaholism has a positive impact on the collective organizational engagement of TMT members for two reasons.

On the one hand, as a symbol of power within the organizational hierarchy, CEOs serve as role models with their words and deeds (Peterson et al., 2009). Workaholic CEOs have a substantial behavioral and cognitive investment in work. Through daily work interactions, workaholic CEOs may convey their work devotion to their TMT members and simultaneously set examples to show how to work hard. In this manner, TMT members would be motivated to increase their devotion to work, thus elevating TMTs' collective organizational engagement. On the other hand, workaholic CEOs work excessively hard themselves and tend to set high standards for their TMT members, which would push TMT members to complete tasks actively and become collectively engaged at work. Therefore, we anticipate that workaholic CEOs will encourage TMTs' collective organizational engagement.

When TMT members collectively engaged in their work, they would proactively put forward their solutions to improve overall organizational efficiency and exhibit behaviors that benefit the firm, ultimately reflecting on the firm's performance. Moreover, with a high level of collective organizational engagement, TMT members are more likely to set aside their own self-interests to pursue organizationally valued objectives, thus promoting firm performance (Barrick et al., 2015). Prior research has found significant positive relationships between collective organizational engagement and firm performance (Barrick et al., 2015). Therefore, we propose that guided by workaholic CEOs, TMT members will be able to achieve higher levels of collective engagement, ultimately attaining higher levels of firm performance. This mediation process is consistent with upper echelons theory's proposal that CEO attributes have important effects on firm outcomes via TMT functioning (Hambrick and Finkelstein, 1996). In sum, we propose:

*Hypothesis 1: TMTs' collective organizational engagement mediates the effect of CEO workaholism on firm performance*.

### The Moderating Role of TMT Power Distance

Upper echelons theory explicitly acknowledges that the strength of CEO attributes' influence would be affected by TMT characteristics (Hambrick and Mason, 1984; Hambrick and Finkelstein, 1996). Specifically, if TMT members conform to the CEO, they are more likely to be influenced by CEOs' attributes. In this case, we further propose that higher TMT power distance would enhance the positive effects of CEO workaholism on TMTs' collective organizational engagement. Power distance is defined as the degree to which individuals accept and believe that organizational power should be distributed unequally (Hofstede, 1980; Farh et al., 2007). Due to frequent interactions and cooperation among TMT members, the TMT as a whole would have a shared power distance, reflecting the extent to which the team recognize and respect leaders' authority (Schaubroeck et al., 2007).

TMTs with higher power distance recognize the existence of hierarchy and show deference and obedience to authority figures (Farh et al., 2007). They expect that they are inferior to supervisors in status and are more likely to accept authority directions without question and avoid disagreement with supervisors (Lam et al., 2002). In this case, TMTs with higher power distance have greater respect for CEOs' authority. They may be more open to workaholic CEOs' influence attempts, thus devoting themselves as the CEO expects. Moreover, TMTs high in power distance are more likely to regard leaders as behavioral models (Schaubroeck et al., 2007). They always look toward leaders for directions and tasks completion strategies (Chen et al., 2014). Hence, TMTs with higher power distance may be more willing to emulate workaholic CEOs and follow CEOs' requirements to work hard, ultimately increasing their collective organizational engagement.

On the contrary, TMTs with low power distance believe that they are equal to their CEOs in status, view subordinate disagreement with and criticism of authorities as appropriate, and would negotiate the terms and rules governing them in the organizations (Farh et al., 2007). Since workaholic CEOs always work excessively hard and set high standards for their TMTs (Clark et al., 2016b; Li and She, 2020), TMTs with low power distance tend to evaluate the workaholic CEOs as more stressful. In this case, TMT members may embrace CEOs' workaholic behaviors to a lesser extent, viewing them as less appropriate or even refusing to pattern their behaviors after their workaholic CEOs. As a result, the collective organizational engagement level of the TMT will decrease. We therefore hypothesize the following:

*Hypothesis 2: TMT power distance moderates the positive relationship between CEO workaholism and TMTs' collective organizational engagement, such that this positive relationship is stronger when TMT power distance is high rather than low*.

Given the mediating role of collective organizational engagement between CEO workaholism and firm performance (Hypothesis 1) and the moderating role of TMT power distance on the relationship between CEO workaholism and collective organizational engagement (Hypothesis 2), we also predict that TMT power distance moderates the indirect effect of CEO workaholism on firm performance via collective organizational engagement, thereby demonstrating a pattern of moderated mediation. Thus, we propose:

*Hypothesis 3: TMT power distance moderates the positive indirect effect of CEO workaholism on firm performance via collective organizational engagement, such that the positive indirect effect is stronger when TMT power distance is high rather than low*.

## Method

### Participants and Procedure

We collected our data via an Executive Master of Business Administration (EMBA) Program specifically for state-owned enterprises organized by a university in Beijing, China. With the support of the EMBA center, we randomly selected 10 EMBA classes (class size ranged from 30 to 50), and then invited CEOs in these classes (332 CEOs in total) to participate in our survey. We promised that their participation was voluntary and confidential and each participant would receive a course credit as compensation after completing all surveys. Two hundred and fifty-two state-owned enterprises' CEOs agreed to participate in our survey and provided their firm information and demographic information.

To avoid common method bias (Podsakoff et al., 2003), we collected data from different sources and at different time points. At Time 1, 252 CEOs were required to complete their questionnaires including workaholism and demographic information via an online survey. We also required them to identify the TMT members (including CFOs) whom directly report to them and to provide these TMT members' names and frequently used e-mail addresses. We received 186 CEOs' surveys, with a response rate of 73.8%. At Time 2 (1 month after Time 1), we sent e-mails to invite these TMT members (excluding CFOs) to fill out the online survey including collective organizational engagement and demographic information. We sent out 1,116 online questionnaires and received 756 of them with a response rate of 67.7%. At Time 3 (1 month after Time 2), online questionnaires including the dependent variable (i.e., firm performance) were distributed to CFOs who work with CEOs in the same company. We invited 186 CFOs and 122 of them responded to our request with a response rate of 65.6%. The three different questionnaires, for CEOs, TMT members, and CFOs, were separately labeled with code numbers to match them correctly.

After matching the three rounds of survey data, we obtained 122 matched CEO-TMT responses (122 state-owned enterprises' CEOs and 676 TMT members). To test dropout bias, we conducted the analysis approach proposed by Goodman and Blum (1996). The coefficients of demographic variables (participants' gender, age, education, and tenure) in multiple logistic regression were all non-significant (*p* > 0.05), suggesting that data were missing completely at random. Of the 122 CEOs, 71 were male. Their average age was 47.6 years (*SD* = 7.33), and average organizational tenure was 12.8 years (*SD* = 5.28). 72.2% of them had a bachelor's degree, and the rest had a master's degree or higher. The TMT ranged in size from 5 to 13 individuals (*mean* = 8.01; *SD* = 2.34). As for TMT members, the average age was 42.7 (*SD* = 6.45), average organizational tenure was 7.4 years (*SD* = 4.67), and 69.7% were male. Moreover, 67.3% of them had a bachelor's degree, and the rest had a master's degree or higher.

### Measures

All the variables we measured were from validated scales, and we followed the Brislin's (1986) suggestion to create the Chinese version of our measures. Specifically, all measures were independently translated from English into Chinese and then translated back into English by two bilingual research assistants. After that, we conducted a pilot study of 10 participants (excluded from the main survey) to check the comprehensibility and applicability of our measures and then made corresponding modifications. All items were assessed on a 7-point Likert-type scale ranging from 1 (strongly disagree) to 7 (strongly agree).

#### CEO Workaholism

CEOs rated 10 items on the workaholism scale developed by Schaufeli et al. (2009). A sample item is: “I seem to be in a hurry and racing against the clock.” The Cronbach's alpha coefficient was 0.92.

#### TMT Collective Organizational Engagement

TMT members rated 5 items on the scale developed by Barrick et al. (2015). A sample item is: “In TMT, my coworkers and I tend to be highly focused when doing our jobs.” The Cronbach's alpha score was 0.91. Agreement among TMT members' ratings shows a mean *RWG* of 0.88, an *ICC (1)* of 0.16 (*p* < 0.001), and an *ICC (2)* of 0.52, suggesting that it was appropriate to aggregate this measure to the team level.

#### TMT Power Distance

TMT power distance was measured using the 6-item scale developed by Farh et al. (2007). A sample item is: “CEOs should make most decisions without consulting TMT members.” Following previous literature (e.g., She et al., 2019), we aggregated TMT members' ratings to the team level via the additive model (Chan, 1998). The Cronbach's alpha score was 0.90. Agreement among TMT members' ratings shows a mean *RWG* of 0.85, an *ICC (1)* of 0.20 (*p* < 0.001), and an *ICC (2)* of 0.45, supporting the aggregation of the responses at the team level.

#### Firm Performance

The 5-item scale developed by Lin and Shih (2008) was administered to CFOs to assess firm performance. A sample item was: “Our sales growth rate is better than competitors.” The Cronbach's alpha score was 0.92.

#### Control Variables

We controlled for TMT size and industry codes, to isolate their potential influences on firm performance. Industrial codes of firms fell into seven categories: information, service, manufacture, trade, medical, construction, and other. The codes were operationalized as dummy variables in our subsequent analyses.

### Analysis Strategy

To test our hypotheses, we conducted path analysis by using Mplus 7.0 software (Muthén and Muthén, 2012). To test the mediating effect (Hypothesis 1), we adopted the Monte Carlo parametric bootstrapping procedure (Preacher and Selig, 2012) to estimate the effect size of this indirect effect. In terms of the moderation effect (Hypothesis 2), following Aiken and West's (1991) suggestions, we mean-centered all predicting variables prior to creating product terms, and probed all interactions through a simple slope analysis. We also plotted the interaction effect for 1 SD above and 1 SD below the mean of the moderator (i.e., TMT power distance). As for the moderated mediation effect (Hypothesis 3), we used the moderated path analysis procedure developed by Hayes (2013) and also applied Monte Carlo parametric bootstrapping procedure (Preacher and Selig, 2012) to test the significance of the indirect effect.

## Results

### Multilevel Confirmatory Factor Analysis

We conducted the multilevel confirmatory factor analysis (MCFA) to assess the factor structure of the variables by using Mplus 7.0 software (Muthén and Muthén, 2012). Compared with traditional confirmatory factor analysis, MCFA decomposes the covariance structures into within-group components and between-groups components, which accounts for the nested nature of the data and provides a more accurate parameter estimation (Dyer et al., 2005). The MCFA results showed that our theorized four-factor model displayed an acceptable model fit (χ^2^[336] = 796.92, χ^2^*/df* = 2.37, CFI = 0.93, TLI = 0.92, RMSEA = 0.05, SRMR for Within = 0.07, SRMR for Between = 0.09) and yielded a better model fit than alternative models, including three-factor model 1 (i.e., TMT collective organizational engagement and TMT power distance combined; Δχ^2^ = 258.47, Δ*df* = 6, *p* < 0.001), three-factor model 2 (i.e., CEO workaholism and firm performance combined; Δχ^2^ = 441.49, Δ*df* = 6, *p* < 0.001), three-factor model 3 (i.e., CEO workaholism and TMT collective organizational engagement combined; Δχ^2^ = 579.01, Δ*df* = 6, *p* < 0.001), and three-factor model 4 (i.e., TMT collective organizational engagement and firm performance combined; Δχ^2^ = 177.31, Δ*df* = 6, *p* < 0.001). The detailed fit indexes for all four models are depicted in Table 1.

**Table 1:** Multilevel confirmatory factor analysis results.

**Models**	*****χ^2^*****	***df***	*****χ^2^**/df***	**CFI**	**TLI**	**RMSEA**	**SRMR for within**	**SRMR for between**
Four-factor model: Four factor separated	796.92	336	2.37	0.93	0.92	0.05	0.07	0.09
Three-factor model 1: TMT collective organizational engagement and TMT power distance combined	1055.39	342	3.09	0.87	0.83	0.12	0.13	0.12
Three-factor model 2: CEO workaholism and firm performance combined	1238.41	342	3.62	0.86	0.84	0.07	0.07	0.15
Three-factor model 3: CEO workaholism and TMT collective organizational engagement combined	1375.93	342	4.02	0.86	0.85	0.08	0.12	0.14
Three-factor model 4: TMT collective organizational engagement and firm performance combined	974.23	342	2.85	0.90	0.89	0.06	0.09	0.12

### Hypotheses Testing

The descriptive statistics and the correlations between variables are displayed in Table 2. Table 3 presents the path analysis results. As Table 3 shows, CEO workaholism was significantly related to collective organizational engagement (*b* = 0.25, *p* < 0.05). Collective organizational engagement was significantly related to firm performance (*b* = 0.17, *p* < 0.05), while the relationship between CEO workaholism and firm performance became non-significant (*b* = 0.14, *p* > 0.05), providing support for the mediation effect. We further adopted the Monte Carlo parametric bootstrap procedure (Preacher and Selig, 2012) to estimate the indirect effects of this path. The results showed that the effect size of the indirect effect was 0.04, and the 95% confidence interval from the bootstrap analysis excluded zero [0.01, 0.13]. In summary, hypothesis 1 was supported.

**Table 2:** Means, standard deviations, and correlations.

**Variables**	***Mean***	***SD***	**1**	**2**	**3**	**4**	**5**	**6**	**7**	**8**	**9**	**10**	**11**
1. TMT size	8.01	2.34											
2. Information industry	0.32	0.47	−0.03										
3. Service industry	0.12	0.33	0.05	−0.26^**^									
4. Manufacturing industry	0.09	0.29	0.00	−0.22^*^	−0.12								
5. Trading industry	0.01	0.13	−0.09	−0.09	−0.05	−0.04							
6. Medical industry	0.36	0.48	−0.02	−0.51^***^	−0.28^**^	−0.24^**^	−0.10						
7. Construction industry	0.05	0.22	0.14	−0.16	−0.09	−0.07	−0.03	−0.17					
8. CEO workaholism	5.13	0.86	−0.04	0.17	0.06	−0.02	0.03	−0.25^**^	−0.08	(0.92)			
9. TMT power distance	5.03	1.12	−0.23^*^	0.18^*^	0.02	−0.02	0.11	−0.25^**^	0.12	0.21^*^	(0.90)		
10. TMT collective organizational engagement	4.97	0.97	−0.29^**^	0.05	0.04	0.04	0.05	−0.19^*^	−0.10	0.23^*^	0.36^**^	(0.91)	
11. Firm performance	5.63	0.74	−0.20^*^	0.17	−0.06	−0.01	0.06	−0.19^*^	0.04	0.33^**^	0.18^**^	0.30^**^	(0.92)

*N = 122 CEOs from state-owned enterprises; Digits in parentheses are Cronbach's alpha*.

* *p < 0.05*;

** *p < 0.01*;

*** *p < 0.001*.

**Table 3:** Path analysis results.

**Variables**	**TMT collective**	**Firm**
	**organizational engagement**	**performance**
**Control variables**		
TMT size	−0.11^**^ (0.04)	−0.03 (0.03)
Information industry	0.28 (0.42)	0.04 (0.33)
Service industry	0.36 (0.46)	−0.14 (0.36)
Manufacturing industry	0.58 (0.49)	−0.10 (0.38)
Trading industry	0.33 (0.74)	0.09 (0.58)
Medical industry	0.23 (0.43)	−0.10 (0.33)
Construction industry	0.82 (0.55)	0.25 (0.43)
**Independent variable**		
CEO workaholism	0.25^*^ (0.10)	0.14 (0.07)
**Mediator**		
TMT collective organizational engagement		0.17^*^ (0.07)
**Moderator**		
TMT power distance	0.21^*^ (0.10)	
**Interaction term**		
CEO workaholism × TMT power distance	0.16^*^ (0.07)	
*R^2^*	0.31	0.24

*N = 122 CEOs from state-owned enterprises; Unstandardized coefficients are reported; Digits in parentheses are standard errors*.

* *p < 0.05*;

** *p < 0.01*.

**Figure 2:**
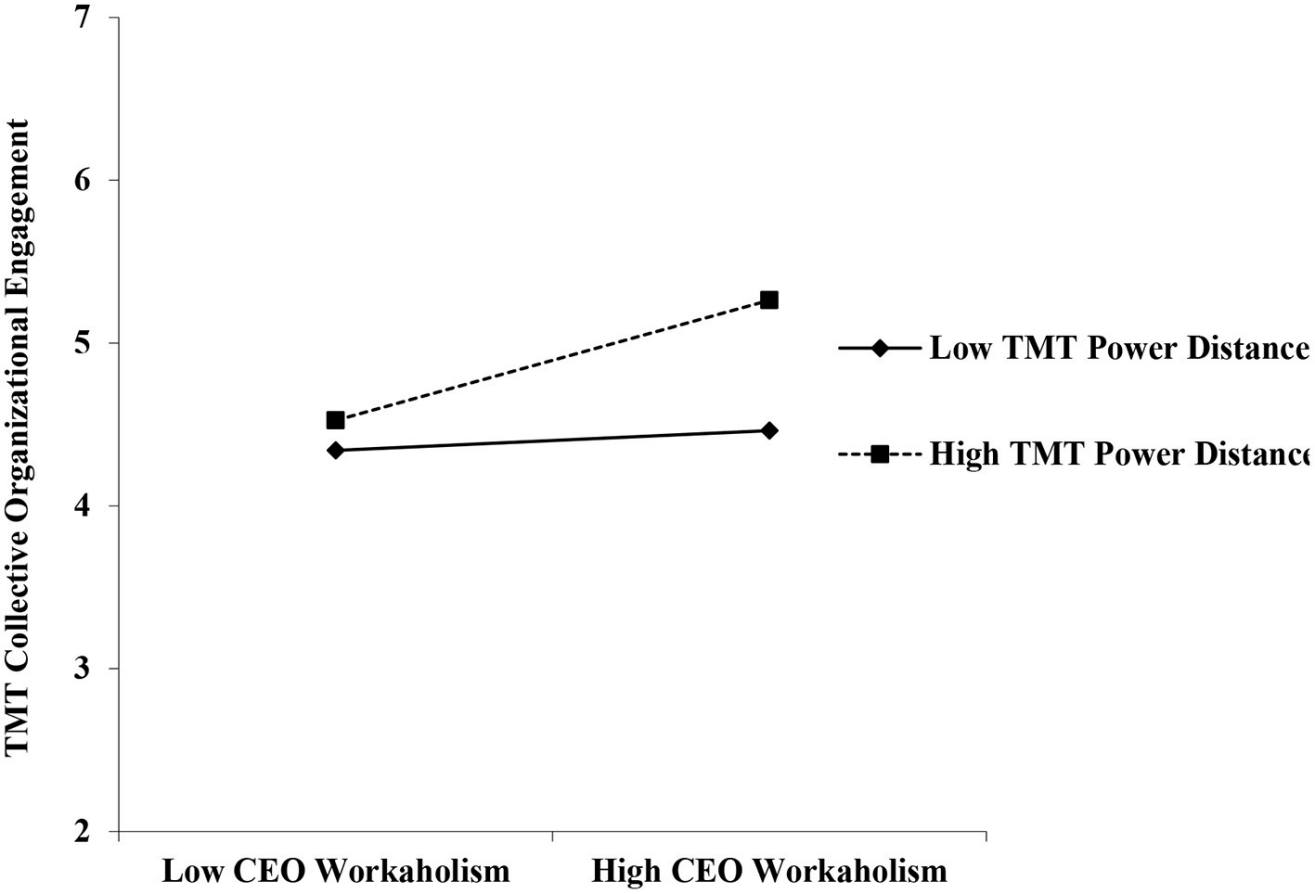
Moderating effect of TMT power distance on the relationship between CEO Workaholism and TMT collective organizational engagement.

As shown in Table 3, the interaction term of CEO workaholism and TMT power distance was positively and significantly related to collective organizational engagement (*b* = 0.16, *p* < 0.05). Furthermore, we followed Aiken and West's (1991) procedures in presenting the pattern of the moderating effect in Figure 2. The simple slope test revealed that CEO workaholism was positively and significantly related to collective organizational engagement when TMT power distance was high (*simple slope* = 0.43, *t* = 2.83, *p* < 0.01), but this relationship was attenuated and non-significant at a low level of TMT power distance (*simple slope* = 0.07, *t* = 0.52, *p* > 0.05). Thus, hypothesis 2 was supported.

To test hypothesis 3, we estimated the indirect effects of CEO workaholism on firm performance at both low and high levels of TMT power distance. When TMT power distance was higher (1 SD above the mean), the indirect effect of CEO workaholism on firm performance through collective organizational engagement was significant (*indirect effect* = 0.07, 95% CI = [0.02, 0.17]); when TMT power distance was lower (1 SD below the mean), the indirect effect was non-significant (*indirect effect* = 0.01, 95% CI = [−0.03, 0.13]). Furthermore, the bootstrapping results showed that the moderated mediation effect was 0.06, and the 95% confidence interval excluded zero [0.01, 0.15]. Taken together, these results supported hypothesis 3.

Although we measured variables from multiple sources at different time points, the research design was cross-sectional in nature, which may raise the concern of reverse causality. It is possible that firm performance is positively related to CEO workaholism. When organizations have excellent performance, to maintain organizational success, the CEOs are more likely to work hard, thus exhibiting workaholic tendency. In this case, workaholic CEOs would motivate their TMT members to exert more efforts for the organizational goals and objectives, which in turn leads to higher collective engagement. Thus, we conducted a supplemental analysis to test the alternative model (i.e., firm performance → CEO workaholism → TMT collective engagement). The results showed that our hypothesized model (i.e., firm performance as the outcome; χ^2^[2] = 11.32, χ^2^*/df* = 5.66, CFI = 0.94, TLI = 0.88, RMSEA = 0.10, SRMR = 0.06) had a better model fit index than the alternative model (i.e., TMT collective engagement as the outcome; χ^2^[2] = 23.84, χ^2^*/df* = 11.92, CFI = 0.82, TLI = 0.77, RMSEA = 0.14, SRMR = 0.11), mitigating the concern of reverse causality to some extent.

## Discussion

Based on upper echelons theory, this study examines how and under what conditions CEO workaholism affects firm performance. Analysis of data from 122 CEOs and 676 TMT members from state-owned enterprises yields the following findings. CEO workaholism is positively related to TMT's organizational collective engagement, which subsequently improves firm performance. Further, TMT power distance moderates the relationship between CEO workaholism and TMT's organizational collective engagement. Through these investigations, the current study makes several theoretical and practical contributions for future research.

### Theoretical Contributions

The current research makes three significant theoretical contributions. First, this study reveals the relationship between CEO workaholism and firm performance, which enriches research on the effectiveness of leader workaholism at firm level. Although studies increasingly highlighted the effects of individuals' workaholism on themselves or their partners (Clark et al., 2016a), there is little evidence about the effectiveness of leader workaholism. To the best of our knowledge, only a few studies have discussed the effects of leader workaholism (Clark et al., 2016b; Pan, 2018; Li and She, 2020), but these studies have mainly focused on its impact on the individual level (i.e., the employee level). The impact of workaholic leaders at firm level has remained unclear. Through empirical analysis, this study preliminarily examines the impact of CEO workaholism on firm performance, which thus extends research from the individual level to the firm level and responds to Clark et al.'s (2016b) call for a more refined view of the effects of leader workaholism.

Second, drawing from upper echelons theory, our study illuminates the “black box” by empirically underscoring the importance of TMTs' collective organizational engagement as a mediator between CEO workaholism and firm performance. As Peterson et al. (2003) claimed, CEO attributes would greatly affect TMT functioning, and then influence more distal, firm outcomes. In this regard, we further explore TMTs' collective organizational engagement as the key transmission mechanism, which highlights the importance of TMT functioning and helps better explain how workaholic executives ultimately affect firm performance. In doing so, we also respond to the call for more examinations about the specific mechanisms through which leader workaholism impacts subordinates (Li and She, 2020). In addition, prior research has focused almost exclusively on the negative effects of workaholism (i.e., Robinson et al., 2001; Schaufeli et al., 2008; Dordoni et al., 2019; Langseth-Eide, 2019), despite Ng et al.'s (2007) claims stressing the potential positive effects of workaholism. Hence, we extend past research by suggesting that workaholic CEOs can motivate their TMT members and thus promote firm performance to some extent.

Third, drawing on upper echelons theory, this study reveals the boundary conditions of the effects of CEO workaholism on TMTs' collective organizational engagement. Specifically, the results showed that the positive relationship between CEO workaholism and TMTs' collective organizational engagement was stronger among TMTs with high power distance. Our consideration of TMTs' shared value (i.e., power distance) contributes important information about the contingency factors that shape the effects of CEO workaholism. Also, it enriches the understanding of how certain contextual and personal factors influence team members' perceptions and responses to a workaholic leader. Extrapolating from this point of view, future research can examine other boundary conditions associated with CEO workaholism to understand its influence fully. For example, work centrality, reflecting how individuals identify with their work roles (Paullay et al., 1994), may enhance the positive effect of CEO workaholism. TMT members with high levels of work centrality regard work as an important part of life (Paullay et al., 1994), thus being more likely to accept and recognize workaholic CEOs in terms of working attitude and value. In this case, they would be more willing to follow workaholic CEOs, resulting in higher collective engagement.

### Practical Implications

This study also carries some significant implications for practice. First, our study indicates that workaholic CEOs devote themselves at work, which could set examples to motive TMT members work hard. In this case, as the representative core of organizations, CEOs are recommended to show more dedication to and absorption in their work and to demonstrate positive working attitudes among their TMT members, thus encouraging TMT members to strive for success and make more contributions to organizations. However, previous studies have also demonstrated the potential negative effects of workaholism on individuals themselves and their families (i.e., Langseth-Eide, 2019). Therefore, it should also be noted that workaholism tendency may have harmful influence for CEOs. We thus suggest that those potential workaholic CEOs always be vigilant in detecting their own workaholism levels and learn how to work smartly rather than work excessively. Besides, organization-sponsored training that includes learning from smart working styles through case analyses, role play, and business simulation would also be helpful in this regard.

Second, our study found that TMTs' collective organizational engagement is positively related to firm performance. This highlights the need for firms to enhance employee engagement at the firm level. Specifically, besides the role modeling effects of workaholic CEOs, firms could offer some team-building activities, such as role play or simulation games, and adopt various managerial practices to stimulate TMT members' engagement as a whole. Third, our findings suggest that workaholic CEOs are more beneficial when TMTs have high levels of power distance. This indicates that firms should be concerned about the match between the CEOs' working style and specific situations (i.e., TMT members' shared values). If the CEO is highly workaholic, it is relatively ideal to select high power distance TMTs working with him or her, because those TMT members may be more respectful and willing to accept the requirements of workaholic CEOs.

### Limitations and Future Directions

Despite the implications discussed above, some limitations should be noted. First, we employed the sampling method that TMT members were nominated by CEOs to participate in our survey. Although this approach has been applied in previous literature (e.g., De Hoogh and Den Hartog, 2008), it may introduce possible selection biases (Marcus et al., 2017). For example, CEOs might intend to invite TMT members who have close working or personal relationship with them. Therefore, we recommend future studies to verify our findings by using different sampling methodologies.

Second, although we applied multi-time and multi-source survey design, the data were cross-sectional in nature. Thus, our study warrants the concern of reverse causality. Accordingly, we conducted a supplementary analysis which showed that the reverse relationship was less likely. Nevertheless, we still cannot draw definitive causal inferences from the current results. Therefore, we recommend future research to test our model more rigorously through laboratory or field experiments. A longitudinal design could also be adopted to strengthen the possibility of inferring causality.

Third, our study was conducted in the Chinese context, where individuals are characterized by high power distance and tend to accept and emulate the authority than those in Western context (Hofstede, 2001; Sarfraz et al., 2020). As such, our results may provide an optimistic estimate of the moderating effect of power distance. In addition, past research has demonstrated that heavy work involvement is highly regarded in China, with deep roots in Confucian values (Hu et al., 2014). TMT members might tend to regard CEO workaholism as professional dedication, which might affect their attitudinal and behavioral responses to the CEO. Therefore, we suggest future research to conduct cross-cultural studies to examine the generalizability of our findings and to explore whether the effectiveness of workaholic CEOs would differ among various cultural contexts.

## Conclusion

Drawing on upper echelon's theory, the current study found that CEO workaholism facilitated firm performance through TMT collective organizational engagement when TMT had higher power distance. Our findings offer preliminary but important insights regarding *when* and *how* CEO workaholism is likely to be positively related to firm performance. We hope that our research advances the understanding of the potential positive effects of leader workaholism and motivates additional examinations of the effects of leader workaholism in organizations so that this complex phenomenon can be better understood.

## Data Availability Statement

The raw data supporting the findings of this study will be made available by the authors to qualified researchers upon reasonable request.

## Ethics Statement

The studies involving human participants were reviewed and approved by Renmin University of China. All subjects gave written informed consent in accordance with the Declaration of Helsinki. The protocol was approved by the Ethics Committee of Renmin University. The patients/participants provided their written informed consent to participate in this study.

## Author Contributions

ZS and QL led the literature review, research design, and paper drafting work. JZ made contributions in data analysis and paper revision. All authors contributed to the article and approved the submitted version.

## Funding

This research was supported by China Postdoctoral Science Foundation (Project ID: 2020M680798), National Natural Science Foundation of China (NSFC, Project ID: 72002108), the Fundamental Research Funds for the Central Universities (Project ID: 63212126), and the Fundamental Research Funds for the Central Universities and the Research Funds of Renmin University of China (Grant No. 21XNLG07).

## Conflict of Interest

The authors declare that the research was conducted in the absence of any commercial or financial relationships that could be construed as a potential conflict of interest.

## Publisher's Note

All claims expressed in this article are solely those of the authors and do not necessarily represent those of their affiliated organizations, or those of the publisher, the editors and the reviewers. Any product that may be evaluated in this article, or claim that may be made by its manufacturer, is not guaranteed or endorsed by the publisher.
